# Interactive actuation of multiple opto-thermocapillary flow-addressed bubble microrobots

**DOI:** 10.1186/s40638-014-0014-3

**Published:** 2014-10-23

**Authors:** Wenqi Hu, Qihui Fan, Aaron T Ohta

**Affiliations:** 1 Department of Electrical Engineering, University of Hawaii at Manoa, 2540 Dole Street, Holmes Hall 483, Honolulu, HI 96822, USA; 2 Department of Mechanical Engineering, University of Hawaii at Manoa, 2540 Dole Street, Holmes Hall 300, Honolulu, HI 96822, USA

**Keywords:** Microrobot, Bubble, Thermocapillary flow, Parallel actuation, Interactive control

## Abstract

Opto-thermocapillary flow-addressed bubble (OFB) microrobots are a potential tool for the efficient transportation of micro-objects. This microrobot system uses light patterns to generate thermal gradients within a liquid medium, creating thermocapillary forces that actuate the bubble microrobots. An interactive control system that includes scanning mirrors and a touchscreen interface was developed to address up to ten OFB microrobots. Using this system, the parallel and cooperative transportation of 20-μm-diameter polystyrene beads was demonstrated.

## Background

Microrobots, nontethered microstructures that can physically manipulate objects, are flexible tools for micro-transportation. Various types of microrobots have successfully transported objects such as microbeads [1–4], microgels [5], and single cells [4,5].

Microrobotic transportation has two advantages when compared to tools such as optical tweezers, optically induced dielectrophoresis (ODEP), and micromanipulators. First, microrobotic transportation does not rely on the optical properties or chemistry of the target objects and the surrounding medium. In contrast, optical tweezers require a refractive index difference between the objects and medium [6]. The medium properties can also play a role in ODEP manipulation, as the electrical conductivity of the medium can affect the electric field gradients that create the dielectrophoretic force [7]. Secondly, although the most widely used micromanipulators can achieve high velocities during manipulation, their throughput is still limited [8], as this is a serial form of manipulation. Parallel operation of multiple micromanipulators is limited by the working space, due to the macroscale components of the micromanipulators. In contrast, the parallel transportation of microrobots has been demonstrated [1,9–15].

One method for achieving parallel microrobot actuation used frequency-selective microrobots that have mechanical responses at different resonant frequencies [1]. Time-division-multiplexed control signals were sent to two magnetic microrobots to achieve simultaneous, independent locomotion. Other frequency-based addressing methods were applied to three magnetic stick–slip microrobots, independently addressed in parallel [10], and to helical microrobots to enable different swimming velocities [11]. Other methods of controlling multiple magnetic microrobots include having differing magnetization strengths [12] or temporarily disabling the magnetization of the microrobots [13]. Electrostatic actuation was another method used, where slight variations in microrobot dimensions enabled independent addressing [14,15].

However, to simultaneously actuate more microrobots independently by magnetic force or electrostatic force, more complicated control and fabrication methods will be necessary. It is difficult to generate localized magnetic fields at the microscale, so each microrobot has to be fabricated slightly differently in order to be addressed independently using the methods mentioned above.

It is straightforward to create multiple localized optical patterns by using optical elements such as micromirrors [16], spatial light modulators [17], or scanning mirrors [18]. Optical tweezers and OET take advantage of this capability to manipulate multiple targets at the same time [16,17]. This feature of optically addressed systems is also inherent in the opto-thermocapillary flow-addressed bubble (OFB) microrobot system [19,20]. However, unlike ODEP and optical tweezers, the OFB microrobots are gas bubbles in a liquid medium. The actuation of the OFB microrobots is less dependent on the material property and does not require direct laser or electrical field penetration through target objects, limiting the potential for damage to the objects under manipulation.

**Figure 1:**
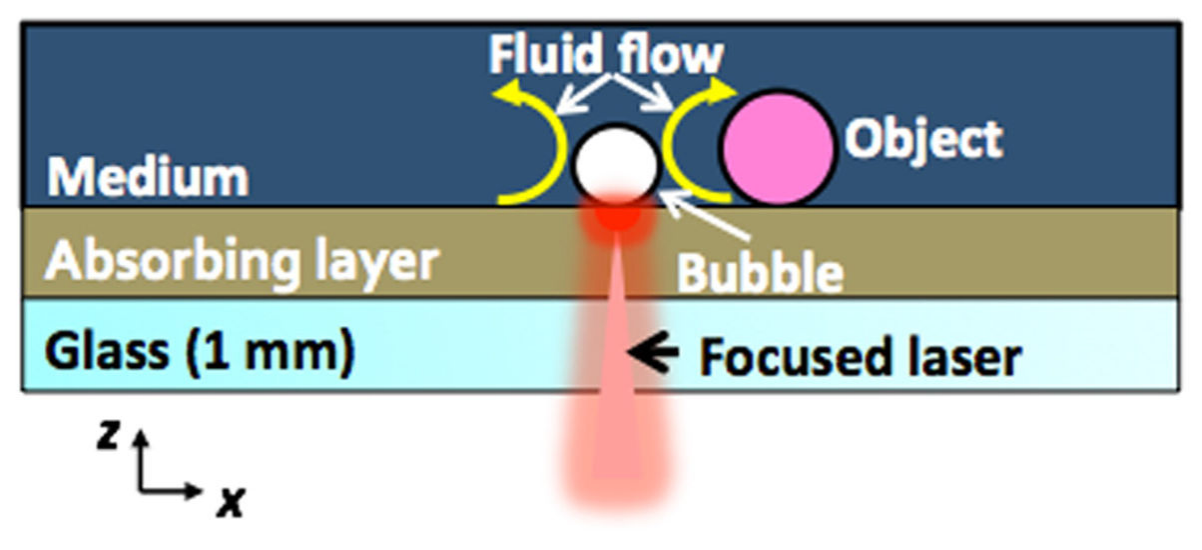
Side view of substrate and liquid medium containing OFB microrobots, showing the opto-thermal actuation mechanism The bubble is stably trapped above the localized hot spot by the temperature gradient created by the laser, and a toroidal thermocapillary flow is formed.

The actuation of the OFB microrobot has been discussed thoroughly in previous publications [19,21]. In summary, a heated region is generated by the selective illumination of the absorbing substrate with a light source. The heated region creates a temperature gradient on the bubble surface, driving a toroidal thermocapillary flow that can be divided into two principal components of interest (Figure 1). The lateral component of the thermocapillary flow is parallel to the substrate and moves the bubble towards the center of the light pattern. The desired effect from this flow component is that the bubble will follow a moving light pattern and will be trapped by a stationary light pattern. Another thermocapillary flow component, perpendicular to the substrate around the bubble perimeter, arises due to the temperature gradient along the vertical direction (*z*-direction).

OFB microrobots have transported microbeads [21], cell-laden hydrogels [19,20], and single cells [21,22]. Although possible, the parallel and independent addressing of more than five OFB microrobots has not been demonstrated. In this paper, multiple OFB microrobots were addressed by an interactive control system with a scanning mirror. Up to ten OFB microrobots are addressed and configured. In addition, four OFB microrobots were used to simultaneously pattern microbeads in hydrogel prepolymer. Microbeads were also handled by four OFB microrobots to demonstrate its cooperative working ability.

## Methods

**Figure 2:**
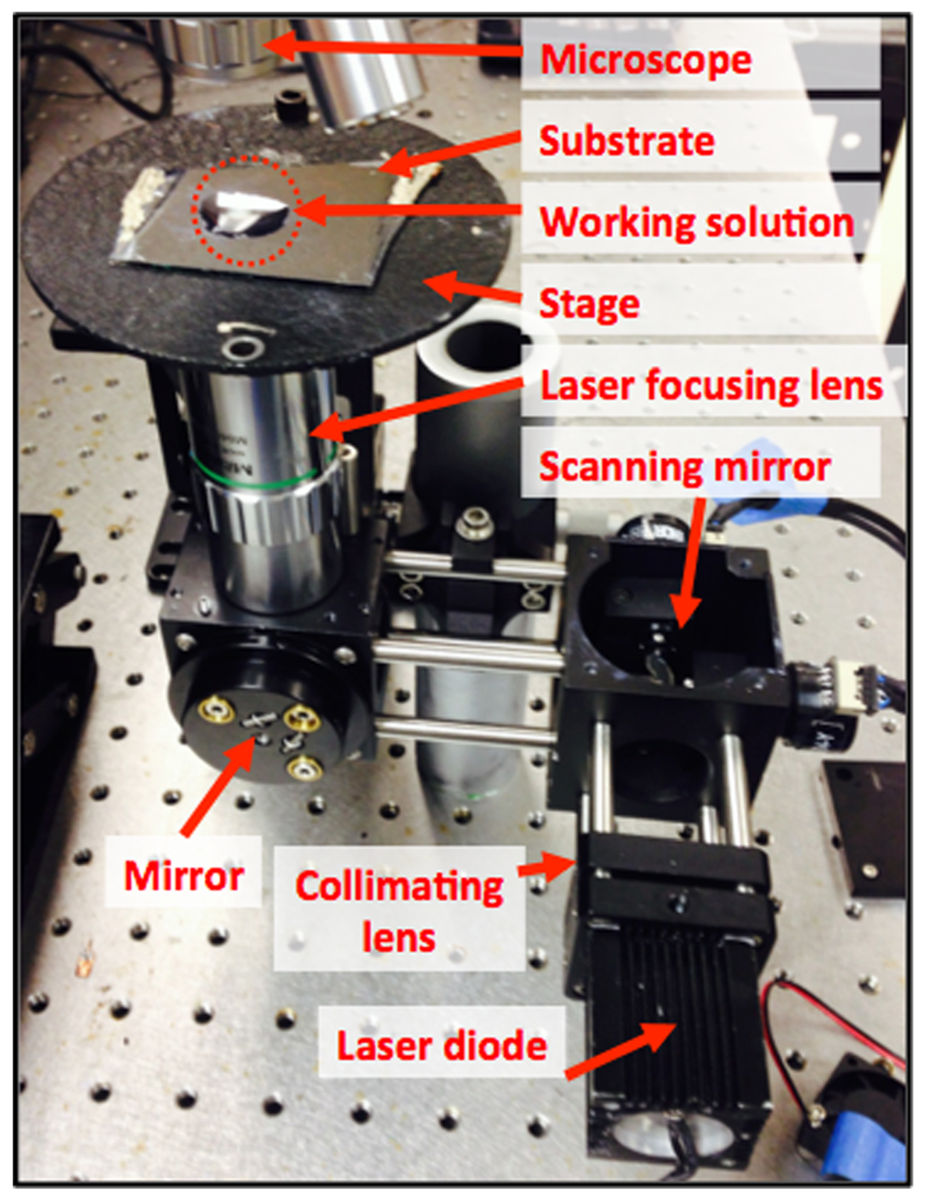
The optical setup of the interactive OFB microrobot system The cross section of the substrate and working solution (marked by dotted circle) is shown in Figure 1.

The optical setup of the OFB microrobot is depicted in Figure 2. A 980-nm laser (Laserlands 980MD-0.4 W-BL, Laserlands, Wuhan, China) was focused to a spot width of 4.4 μm by a 0.42-N.A. ×20 objective lens (Mitutoyo, Takatsuku, Kawasaki, Japan) onto the absorbing substrate. Unlike previous setups for single OFB microrobots [19,21], a dual-axis scanning mirror system (Newport GVSM002, Newport Corporation, Irvine, CA, USA) is integrated into the light path to direct the laser to different locations on the substrate.

The OFB microrobot actuation takes place on an absorbing substrate. The absorbing layer on the substrate is made of a layer of indium tin oxide (ITO) that is 100 nm in thickness, topped with a layer of 1-μm-thick amorphous silicon (α-Si). This absorbing layer is able to convert light energy into heat. The ITO layer also serves as an adhesion layer for the α-Si.

**Figure 3:**
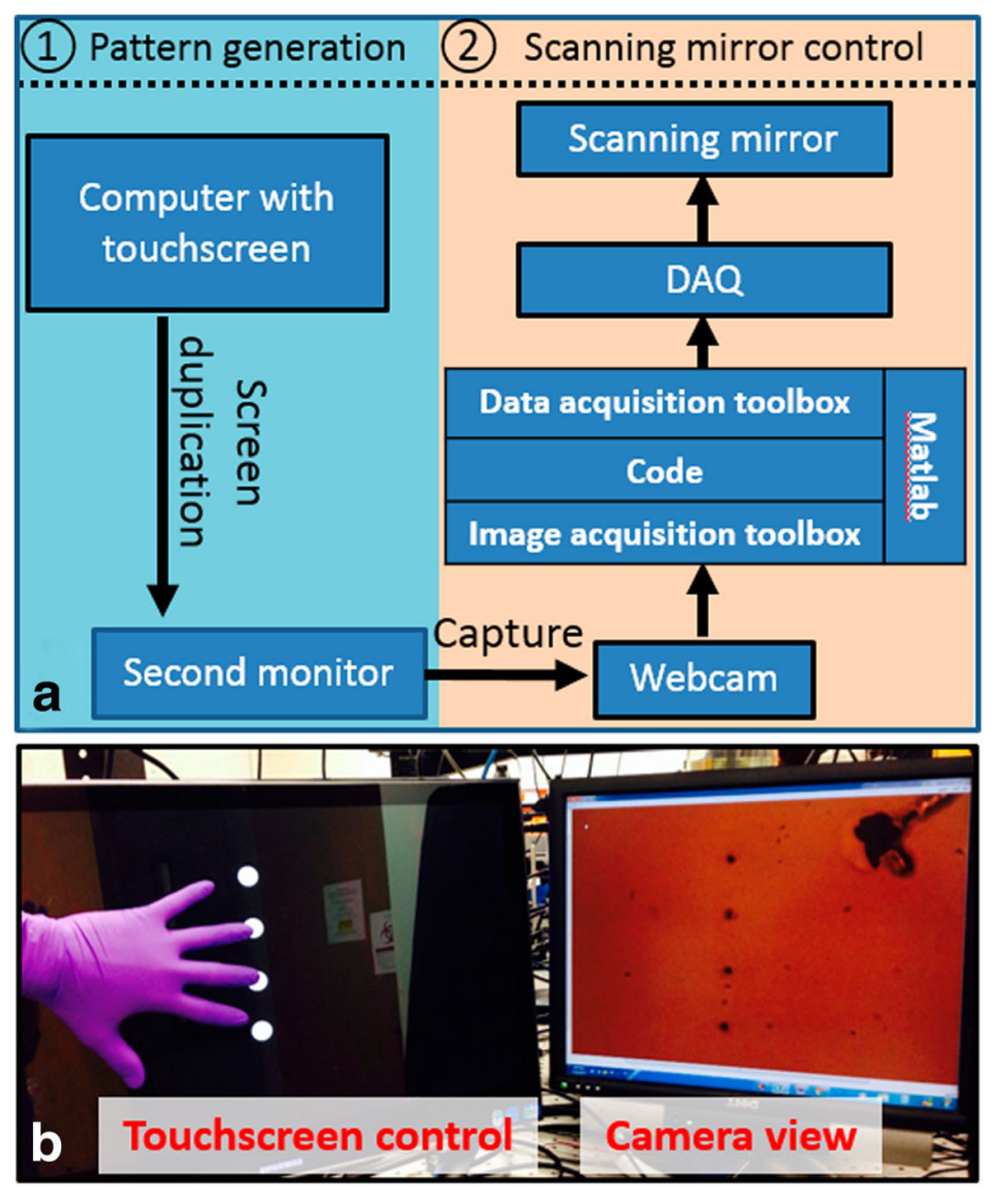
Interactive control system and interface **(a)** The interactive control system of OFB microrobot, showing the two subsystems: pattern generation and scanning mirror control. **(b)** The interactive control interface. The positions of the four OFB microrobots visible in the camera view of the working space were controlled by the four white spots on the touchscreen.

The control system of the OFB microrobot is made of two subsystems: pattern generation and scanning mirror control (Figure 3a). The pattern generation subsystem uses a touchscreen computer (Samsung Series 7, Samsung Electronics, Suwon, South Korea) as the interface to the system operator. An interactive control application was written using the simple multi-touch (SMT) library of the Processing programming tool [23]. The application created for the OFB microrobot control system has the ability to create, reposition, and remove light patterns within a set workspace, according to the touch input of the operator. These light patterns control the movement of the microrobots and appear on the touchscreen as white circles on a black background (Figure 3b). The touchscreen display was aligned with a live-view camera feed from the microscope, enabling the operator to simultaneously control the light sources while receiving instant visual feedback. The display on the touchscreen was duplicated on a second monitor, which is captured by a webcam (Logitech C170, Logitech International S.A., Lausanne, Switzerland) attached to the scanning mirror control subsystem.

The centerpiece of the scanning mirror control system is a custom script that uses the MATLAB Image Acquisition Toolbox to determine the positions of the light patterns on the second computer monitor. The *x* and *y* positions of the light patterns are recorded using units of pixels, which are converted to analog voltages by the MATLAB script. Subsequently, the script uses the MATLAB Data Acquisition Toolbox to queue the analog voltages into a data acquisition unit (DAQ; National Instruments USB-3653, National Instruments Corporation, Austin, TX, USA). The DAQ outputs the voltages to the control circuits of the scanning mirror system, thereby controlling the position of the mirrors. Thus, the scanning mirrors are adjusted to the correct angles to redirect the laser to the corresponding position on the substrate.

The scanning mirror finishes scanning through all the points of the light patterns in 0.05 s per cycle of the control script, independent of how many points there are in the cycle. Bubbles can be generated immediately in the liquid medium by the laser illumination, and longer illumination durations lead to larger bubble sizes [21]. To control the bubble size, the heat generation at each location can be further controlled by pulsing the laser at various frequencies and duty cycles. This is controlled by a TTL signal provided by a function generator (Agilent 33220A, Agilent Technologies, Santa Clara, CA, USA). In the experiments presented here, the laser pulse frequency was 400 Hz, and the laser pulse width was 100 μs. This resulted in bubbles with diameters ranging from 7 to 10 μm, with an average diameter of 8 μm. The bubbles do not collapse during the experiments, as the laser pulse rate is rapid enough to maintain the bubble. However, if the laser is switched off, the bubble will collapse within 2 s due to the Laplace pressure. Under these actuation conditions, the OFB microrobots can move up to 500 μm/s.

The current control system requires two computers, one running the Processing program for the pattern generation subsystem and one running the MATLAB script for the scanning mirror control subsystem, as well as an extra monitor. It is currently nontrivial to create MATLAB scripts that will support touchscreen input, so this hardware workaround was implemented to take advantage of the image processing of MATLAB and the multi-touch library of Processing. This control system can be easily replicated by any research group in the microrobotic or micromanipulation research areas.

## Results and discussion

### Control of multiple OFB microrobots

**Figure 4:**
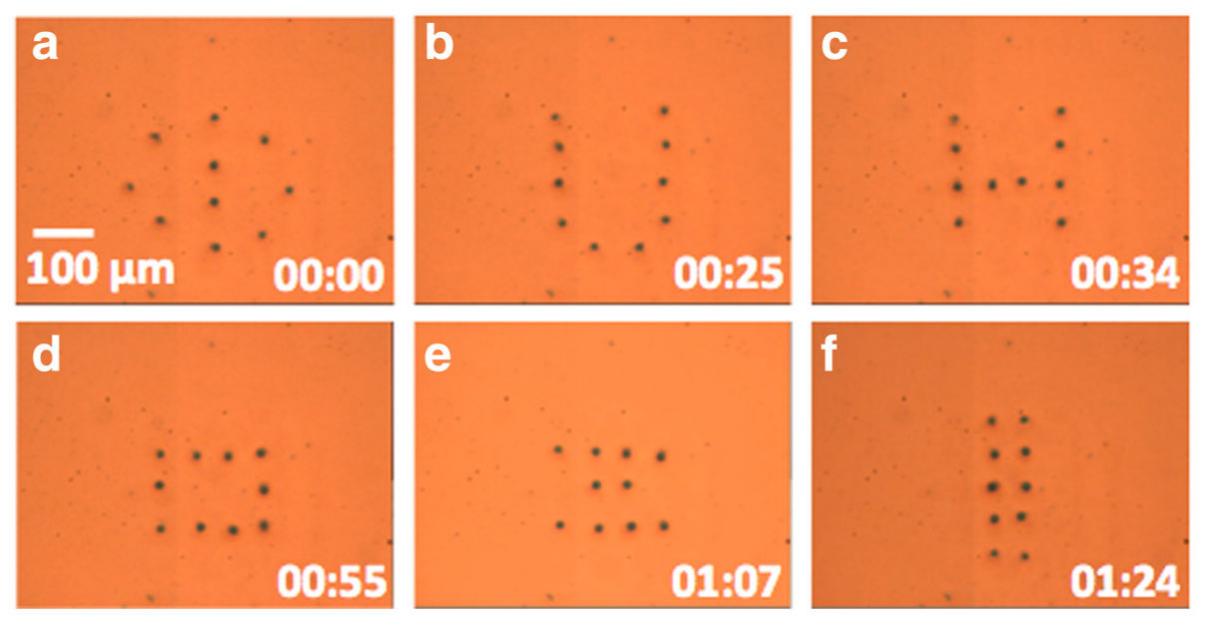
Ten OFB microrobots were sequentially configured into different patterns (a–f) using the interactive control system The time stamp format is minutes: seconds.

Using the interactive microrobot control system described in the previous section, ten bubble microrobots were configured into different patterns, including the letters ‘UH’ and arrays that may be useful in cell patterning [24] (Figure 4). This experiment and all of the following use an aqueous solution that contains 1% agarose prepolymer (type IX, ultra-low gelling temperature). Agarose is a naturally extracted biomatrix that can support cell growth [25], which aligns with the ultimate goal of using this microrobot system to assemble cells.

### Transportation by multiple OFB microrobots

**Figure 5:**
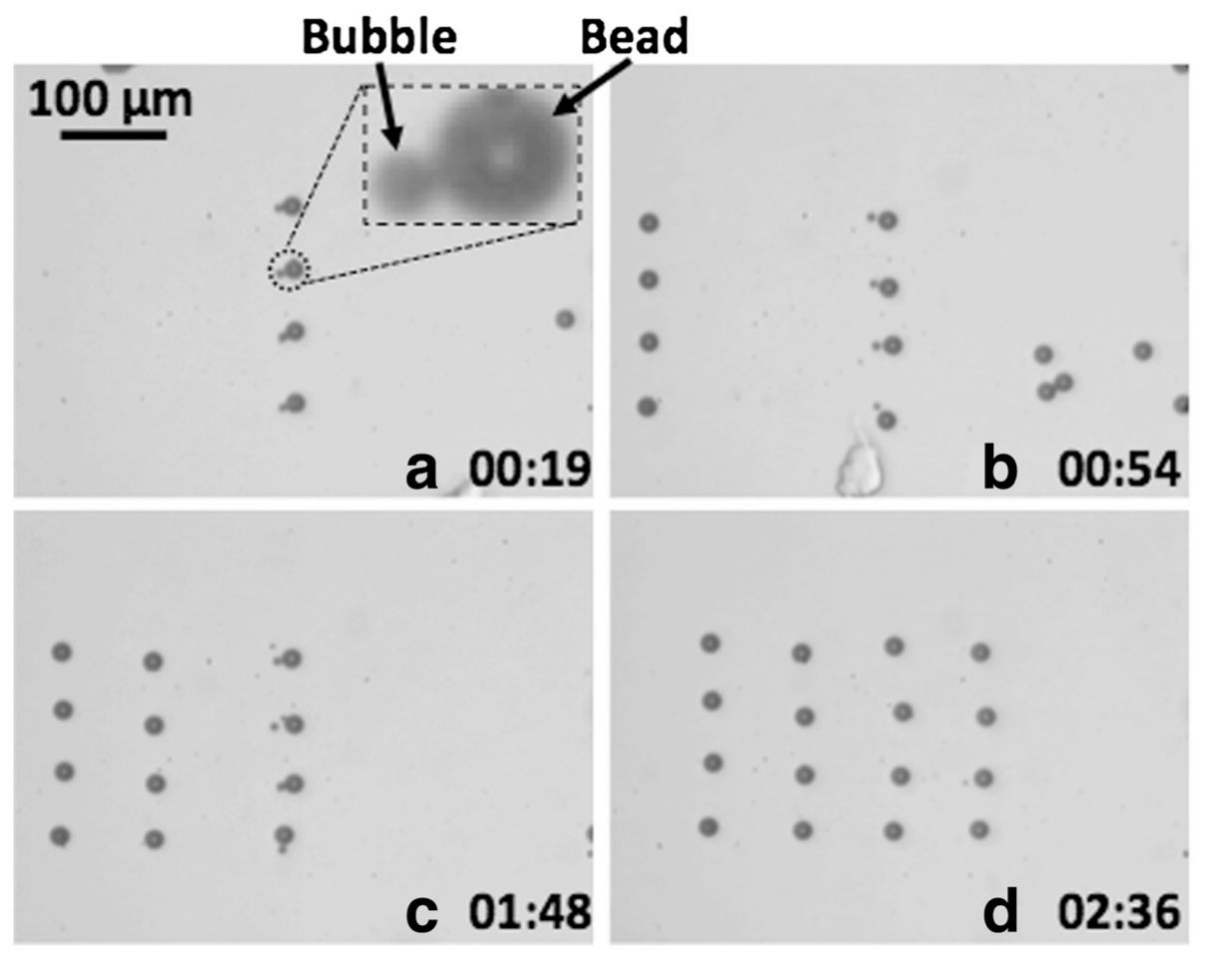
Matrix assembly A 4 × 4 matrix of 20-μm-diameter polystyrene beads in 1% agarose solution was assembled by column of four OFB microrobots. The time stamp format is minutes: seconds. **(a)** Start of assembly. A magnified photo of an OFB microrobot with a bead is shown in the inset. **(b)** The leftmost column of the matrix has been assembled, and the four OFB microrobots are transporting the second column of microbeads. **(c)** Two columns of the matrix have been assembled, and the third column is undergoing transportation. **(d)** The assembled 4 × 4 matrix of beads.

Multiple OFB microrobots can increase the throughput of the micro-transportation. This was demonstrated by assembling a 4 × 4 matrix of 20-μm-diameter polystyrene beads using a column of four OFB microrobots (Figure 5). To assemble each column of beads, the four microrobots first pulled four beads close to each microrobot, so that each microrobot was transporting a single bead. Then, the four microrobots moved to the patterning sites with the beads following behind them (Figure 5a). Finally, the laser was turned off, terminating the thermocapillary flow and releasing the beads from the microrobots. The process was then repeated three more times, completing the 4 × 4 matrix. The entire assembly process was completed in only 2.5 min, less than half the time needed by a single OFB to build the 3 × 3 cell matrix in the same medium (approximately 6 min) [21].

**Figure 6:**
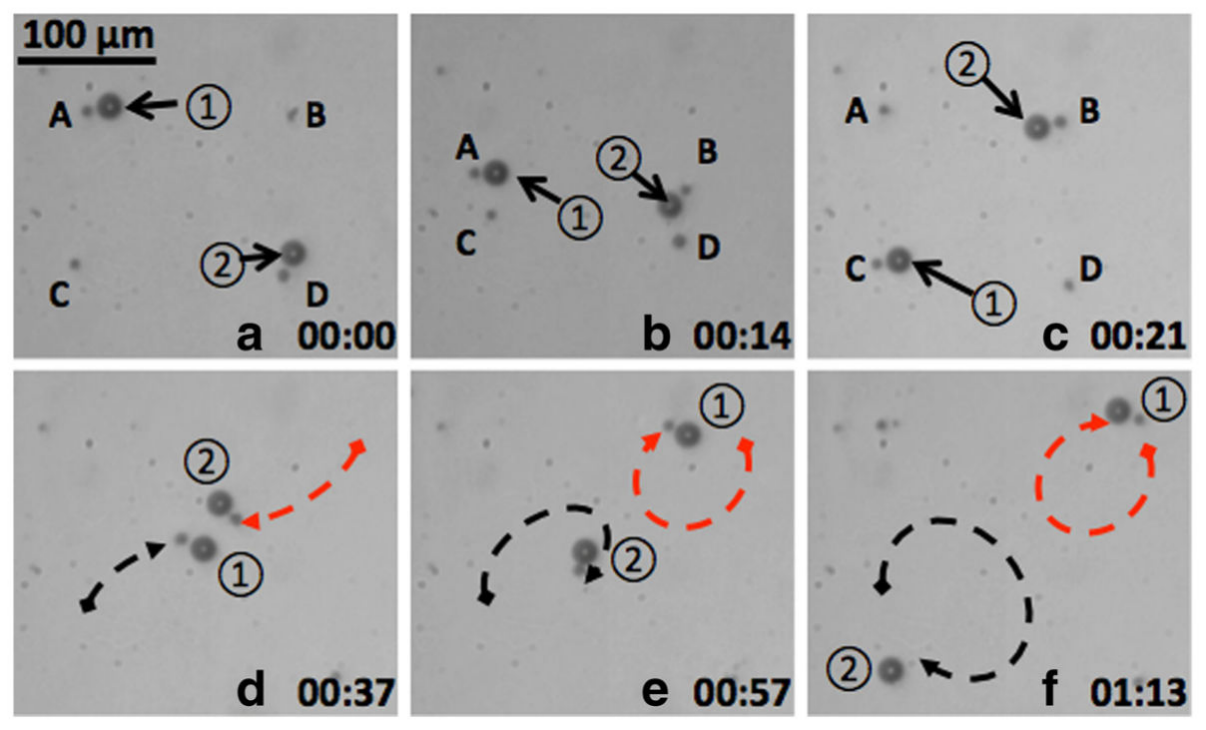
Two 20-μm-diameter beads were passed between four independently controlled OFB microrobots The four OFB microrobots are marked as A, B, C, and D. The two 20-μm beads are marked by 1 and 2. The time stamp format is minutes: seconds. **(a–c)** OFB microrobot A transferred the no. 1 bead to microrobot C; at the same time, microrobot D transferred the no. 2 bead to microrobot B. **(d)** The beads are transported to the center of the field of view. **(e)** The beads were exchanged between microrobots B and C. **(f)** The OFB microrobots then transported the exchanged beads to the side opposite their starting locations in **(d)**.

Moreover, cooperative transportation of micro-objects was also performed. This demonstrates that parallel control of individual microrobots can enable the cooperative use of microrobots to accomplish tasks. Four OFB microrobots were used to perform a cooperative ‘hand-off’ routine, where microbeads were passed between the micro-robots (Figure 6). Two 20-μm beads were first respectively exchanged between the microrobots labeled A and C and the microrobots labeled B and D (Figure 6a,b,c). The two beads were then brought to the center of the screen (Figure 6d) and exchanged between microrobots B and C (Figure 6e). At last, as shown in Figure 6f, the beads were brought by the OFB microrobots to the position diagonally opposite to their locations shown in Figure 6c. This experiment demonstrates that multiple microrobots adjacent to a micro-object do not interfere with the manipulation of the object.

## Conclusions

An interactive control system for the OFB microrobot was demonstrated by performing parallel and cooperative assembly of microbeads. This system has the potential to be scaled up even further, increasing the throughput and utility of the OFB microrobotic system.

Although the interactive control system performed well in the experiments described here, the system can be further simplified. The need for the webcam and extra monitor can be eliminated by directly outputting a signal with the light pattern location information from the pattern generation computer to the scanning mirror control computer. A single computer can also accomplish the functionality of the two computers in the current setup, if the MATLAB script is configured to accept input from the touchscreen of its host computer. Moreover, the live camera view of the OFB workspace can also be displayed directly on the touchscreen to make it easier for the operator to co-locate the target objects and the microrobots, similar to the control systems in [26,27].

It can be approximated that increasing the number of microrobots will linearly reduce the completion time of microassembly tasks until a saturation point is reached. Beyond this saturation point, the microrobot density will limit the amount of parallel micromanipulation that can occur. The maximum number of 10-μm-diameter OFB microrobots that theoretically can be generated in the field of view used here (600 μm by 450 μm) is 2,700 microrobots. However, the current system does not approach that limit, so the linear relationship can be used to estimate the impact on task completion time.

The maximum number of microrobots is limited in the current control system by the ability of the human operator to track multiple objects. In these experiments, a maximum of ten microrobots can be controlled by a single operator. In order to scale this system to control a much larger number of microrobots in parallel, automated controls have to be developed. An automated control system can build upon algorithms developed for path planning and macroscale robotic swarm research [28]. In addition to increasing the throughput of microassembly tasks, multiple microrobots can enable certain manipulation tasks, such as studying temporal dynamics of micro-object interactions. In this case, it may be necessary to have several objects moving at various velocities and trajectories relative to one another, which can be achieved by using multiple microrobots.

The micro-object used in the experiments above is a 20-μm polystyrene bead, which is near in size to many biological micro-objects, such as cells. Thus, the results here are expected to be transferrable to these biological micro-objects. As one example, patterning of single mammalian cells into different patterns can benefit research in tissue engineering [29]. When manipulating biological materials, temperature is a concern, as objects such as cells can die at temperatures above the physiological temperature of 37°C. In this system, the maximum temperature is localized to the very center of the laser spot and quickly decreases to a temperature that is safe for cells: the temperature is less than 30.5°C at a distance of 3.5 μm from the center of the laser spot [21]. As the bubble radii in these experiments are approximately 5 μm, this ensures that cells are manipulated at a safe temperature.
